# M2 macrophages‐derived exosomal microRNA-501-3p promotes the progression of lung cancer via targeting WD repeat domain 82

**DOI:** 10.1186/s12935-021-01783-5

**Published:** 2021-02-05

**Authors:** Jie Lei, Peng Chen, Feng Zhang, Na Zhang, Jianfei Zhu, Xiaoping Wang, Tao Jiang

**Affiliations:** 1 Department of Thoracic Surgery, The Second Affiliated Hospital of Air Force Medical University, 569 Xin Si Road, Xi’an, 710038 Shanxi China; 2 Department of Oncology, The Second Affiliated Hospital of Air Force Medical University, Xi’an, 710038 Shanxi China

**Keywords:** Lung cancer, M2 macrophages-derived exosome, MicroRNA-501-3p, WD repeat domain 82

## Abstract

**Background:**

Exosomes are known to transmit microRNAs (miRNAs) to affect cancer progression, while the role of M2 macrophages-derived exosomes (M2 exosomes) conveying miR-501-3p in lung cancer (LC) remains unknown. We aim to explore the role of exosomal miR-501-3p in LC development via targeting WD repeat domain 82 (WDR82).

**Methods:**

Lung cancer tissue and normal tissue specimens were collected, in which tumor-associated macrophages (TAM) were measured by immunohistochemistry. M2 macrophages were induced and treated with altered miR-501-3p, and then the exosomes were extracted and identified. MiR-501-3p and WDR82 expression in LC tissues and cell liens was determined. The predictive role of miR-501-3p in prognosis of LC patients was assessed, and the proliferation, colony formation ability, invasion, migration and apoptosis of the LC cells were determined. Targeting relationship between miR-501-3p and WDR82 was confirmed.

**Results:**

TAM level was elevated in lung cancer tissues. MiR-501-3p was upregulated while WDR82 was downregulated in LC tissues and cell lines, and the M2 exosomes further upregulated miR-501-3p. M2 exosomes and exosomal miR-501-3p promoted LC cell growth. MiR-501-3p inhibition reversed the effect of M2 exosomes on LC cells. WDR82 was confirmed as a target gene of miR-501-3p.

**Conclusion:**

M2 macrophages-derived exosomal miR-501-3p promotes the progression of LC via downregulating WDR82.

## Background

Lung cancer (LC) is a main reason of cancer death all over the world [1]. There are over 1.8 million newly diagnosed LC cases each year and the mortality is more than 90% [2]. LC is traditionally separated into small cell lung cancer (SCLC) (15% to 25%) and non-small cell lung cancer (NSCLC) (75% to 85%) [3]. The pathogenesis of LC is multifactorial, including environmental and genetic factors, while the occurrence of LC is related to the regulation of tumor suppressor genes and oncogenes [4]. As reported, 70% of LC patients are diagnosed at advanced stages and the five-year survival rate is roughly 16%. Unfortunately, only 15% of LC cases are diagnosed at early stages [5]. Thus, there is an urgent need to explore novel target for the treatment of LC.

Exosomes are natural lipid membrane-enclosed vesicles with diameters arranged from 30 to 150 nm, and can be released and synthesized by multiple cells types [6]. Macrophages are differentiated cells of the mononuclear phagocytic lineage which feature special phenotypic characteristics and particular marker expression, and can promote tumorigenesis [7]. M2 macrophages, also known as alternative-activated macrophages, are known to be able to mediate cancer progression [8]. It has been reported that THP-1 macrophages activated by exosomes derived from lung adenocarcinoma cells promoted LC cell invasion [9]. MicroRNAs (miRNAs) are small (18–22 nucleotides), non-coding RNA molecules which can post-transcriptionally regulate gene expression [10]. Some particular miRNAs, such as miR-143-3p [11] and miR-330-3p [12] have been identified to participate in the progression of LC. As one of the miRNAs, miR-501-3p has been demonstrated to be implicated in the progression of NSCLC [13]. Another study has demonstrated that upregulated miR-501-5p is found in patients with lung adenocarcinoma [14]. Exosomal-enclosed miRNAs have been shown to exert effects in diverse diseases and injuries [15]. For instance, the exosomal miR-21 and miR-4257 and have been revealed to be upregulated in NSCLC [16]. As previously reported, M2 macrophages-derived exosomes (M2 exosomes) upregulated miR-501-3p to promotes progression of pancreatic ductal adenocarcinoma (PDAC) [17]. Moreover, exosomal miR-501 partly contributes to tumorigenic growth in gastric cancer [18], while the role of M2 exosomes transmitting miR-501-3p in LC remains seldom explored. WD repeat domain 82 (WDR82), an integral component of the SETD1A complex, is a key epigenetics-associated factor [19], and it has been unraveled that WDR82 is associated with progression and prognosis in human colorectal cancer [20]. Nevertheless, the binding between miR-501-3p and WDR82 as well as the role of WDR82 in LC remains largely unknown. In this study, we aimed to investigate the role of M2 macrophages-derived exosomal miR-501-3p in the development of LC with the involvement of WDR82, and we supposed that M2 exosomal miR-501-3p could affect LC progression by targeting WDR82.

## Materials and methods

### Ethics statement

Written informed consents were acquired from all patients before this study. The protocol of this study was confirmed by the Ethic Committee of The Second Affiliated Hospital of Air Force Medical University and based on the ethical principles for medical research involving human subjects of the *Helsinki Declaration*.

### Study subjects

Eighty-four pairs of LC tissues and adjacent normal tissues were harvested from LC patients (57 females and 27 males, aged 32–75 years) accepted surgical resection in The Second Affiliated Hospital of Air Force Medical University from June 2012 to June 2014. Patients didn’t undergo radio- or chemo-therapy before the surgery. The clinical information of patients was shown in Additional file 1: Table S1. The tissues were soaked in liquid nitrogen overnight and preserved at − 80℃ on the second day. LC tissues were classified as the standard proposed by the World Health Organization, and the diagnosis was independently confirmed by two pathologists. The follow-up visit lasted for 5 years and ended in June 2019. The overall survival (OS) was calculated from the day of grouping to the day of death from any reason or the last follow-up visit.

### Immunohistochemistry

Frozen sections of LC tissues and normal tissues were rewarmed, fixed in ice-cold acetone, reacted with 0.3% tritonX-100 and blocked with 5% bovine serum albumin. After that, the sections were successively probed with the primary antibody F4/80 (Ab100790, 1:100, Abcam, Cambridge, UK) and horseradish peroxidase-labeled goat anti-rabbit immunoglobulin G antibody (ab6721, 1:1000, Abcam) and developed by diaminobenzidine. Then, the sections were reacted with hematoxylin, dehydrated, and permeabilized. Positive expression was reflected by brown particles. The sections were viewed under five fields of view under a microscope [17].

### Cell culture

LC cell lines (SPC-A1, A549, H1299 and H23) and human normal bronchial epithelial cells HBE (all from Cobioer Co., Ltd., Jiangsu, China) were cultured in Roswell Park Memorial Institute (RPMI)-1640 medium (Thermo Fisher Scientific Inc., MA, USA) containing 10% fetal bovine serum (FBS, Thermo Fisher). THP-1 cells were obtained from American Type Culture Collection (VA, USA) and exosomes were isolated through centrifugation at 100,000*g* at 4 °C overnight. THP-1 cells were cultured in RPMI-1640 medium with 10% phosphate buffered saline, and when the cell confluence reached 90%, they were passaged at 1: 3–4. The cells used were verified by short tandem repeat analysis and were free of mycoplasma contamination. THP-1 cells were treated with 100 ng/mL phorbol myristate acetate (Sigma-Aldrich Chemical Company, MO, USA) for 24 h, and then were treated with 100 ng/mL lipopolysaccharide (LPS, Sigma) and 20 ng/mL interferon γ (IFN-γ) (R&D Systems, MN, USA) for 24 h. After that, THP-1 cells were treated with 20 ng/mL interleukin 4 (IL-4, Peprotech, NJ, USA) for 72 h and polarized into M2 macrophages [21, 22]. The cell surface marker CD206 was identified using flow cytometry.

### Isolation and identification of M2 exosomes

The M2 exosomes were extracted from M2 macrophages as previously described [17], and then a transmission electronic microscope (TEM) was used to identify the exosomes: 20 μL exosomes were added onto a copper mesh for 3 min with the fluid removed, and then were counterstained using 30 μL phosphotungstic acid solution (pH = 6.8) for 5 min. Baked by an incandescent lamp, exosomes were photographed under a TEM. Particle size analysis was performed using nanoparticle tracking analysis (NTA, NS300, Malvern Instruments Ltd., Malvern, UK). The exosome surface markers tumor susceptibility gene 101 (TSG101), CD81 and CD63 were detected using Western blot analysis.

### Cell transfection and grouping

A549 and SPC-A1 cells were treated with M2 exosomes, or transfected with miR-501-3p mimic, miR-501-3p mimic negative control (NC), miR-501-3p mimic NC and pcDNA3.1-WDR82 NC, miR-501-3p mimic and pcDNA3.1-WDR82 NC, or miR-501-3p mimic and pcDNA3.1-WDR82. Also, A549 and SPC-A1 cells were treated with M2 macrophage-derived exosomes and miR-501-3p inhibitor NC, or M2 macrophage-derived exosomes and miR-501-3p inhibitor. Cells transfection (80%-90% cell confluence) was performed with Lipofectamine 2000 reagent (Invitrogen Inc., CA, USA). The plasmids were obtained from GenePharma Co., Ltd. (Shanghai, China). Plasmids (25 pmol) and transfection reagent (10 μL) were mixed, and the mixture (10 pmol/mL) was applied to cell culture. pcDNA3.1-WDR82 (100 nmol/L) was transfected into cells as the NC [17, 23]. A control was set with lung cancer cells with any treatment.

### Cell counting kit-8 (CCK-8) assay

Based on protocols of CCK-8 kits (Beyotime Institute of Biotechnology, Shanghai, China), cells were seeded, cultured for 24 h, and further cultured in 100 μL medium with 10 μL CCK-8 reagent. Absorbance at 450 nm at 14, 48 and 72 h was determined using a Multiscan FC plate reader (Thermo Fisher).

### Colony formation assay

Cells were seeded, cultured for 7 days with medium changed every 3 days, and fixed with paraformaldehyde for 30 s, and then were stained with 0.1% crystal violet solution (Aladdin Holdings Group, Beijing, China) for 30 min. A microscope (TS100, Nikon, Tokyo, Japan) was used for observation of colony formation. Colony formation number was calculated from 10 random fields.

### Flow cytometry

The apoptosis of LC cells was assessed by flow cytometry referring to a publication [24], and the results were determined by a FACSCalibur flow cytometer and the BD FACSCanto™ system software v2.4 (both from BD Biosciences, NJ, USA).

### Scratch test

The scratch test was performed to detect the cell migration ability based on the description in a study [24]. Samples were taken and photographed at 0 and 48 h, and 3 fields of view were selected in each sample to measure the migration rate.

### Transwell assay

Transwell assay was used for detection of migration and invasion as previously described [24]. A microscope was used for cell counting.

### Western blot analysis

Total protein in tissues and cells was extracted. Proteins were conducted with polyacrylamide gel electrophoresis, transferred onto membranes and blocked with 5% skim milk. Then, the proteins were incubated with primary antibodies WDR82 (1: 1000), β-actin (1: 500, OriGene Technologies Inc., Beijing, China), rabbit anti-TSG101, CD63 and CD81 (1: 1000, Abcam) at 4 ℃ overnight, and incubated with relative secondary antibody (1: 2000, Abcam) for 1 h. Image J 1.48u software (National Institutes of Health, MA, USA) was used for protein quantification analysis and glyceraldehyde phosphate dehydrogenase was used as the loading control [17].

### Reverse transcription quantitative polymerase chain reaction (RT-qPCR)

Total RNA was extracted from tissues and cells using RNA extraction kit (Thermo Fisher), and the RNA concentration was measured. Primers used were all synthesized by TaKaRa Biotechnology Co., Ltd. (Liaoning, China) and the sequences were shown in Table 1. Data were calculated using 2^−△△CT^ method [25].

**Table 1:** Primer sequence

Gene	Primer sequence (5′–3′)
miR-501-3p	Forward: GCCGAGAATGCACCCGGGCA
	Reverse: CTCAACTGGTGTCGTGGA
WDR82	Forward: CTCCATCGTGCTCTATGACT
	Reverse: GATGAGGTCCACACCATATT
U6	Forward: CTCGCTTCGGCAGCACA
	Reverse: AACGCTTCACGAATTTGCGT
β-actin	Forward: CAGCACAATGAAGATCAAGA
	Reverse: GATCCACATCTGCTGGAAG

*miR-501-3p* microRNA-501-3p, *WDR82* WD repeat domain 82

### Dual luciferase reporter gene assay

Targetscan (http://www.targetscan.org/vert_72/) was used to predict the binding sites between miR-501-3p and WDR82. WDR82 dual luciferase reporter plasmid (pGL3-WDR82-WT) and mutant of binding sites of WDR82 and miR-501-3p (pGL3-WDR82-MUT) were constructed, which were co-transfected with miR-501-3p mimic and pRLTK (internal reference plasmid expressing Renilla luciferase) into A549 and SPC-A1 cells for 24 h. The reporter plasmids were designed and provided by GenePharma. The luciferase activity was determined [17].

### Statistical analysis

All data analyses were conducted using SPSS 21.0 software (IBM Corp. Armonk, NY, USA). The measurement data were expressed as mean ± standard deviation. The unpaired t-test was performed for comparisons between two groups, one-way analysis of variance (ANOVA) was used for comparisons among multiple groups and Tukey’s post hoc test was used for pairwise comparisons after one-way ANOVA. *P* value < 0.05 was indicative of statistically significant difference.

## Results

### MiR-501-3p is upregulated while WDR82 is downregulated in LC tissues and cells, and high miR-501-3p expression is associated with a poor prognosis

**Fig. 1:**
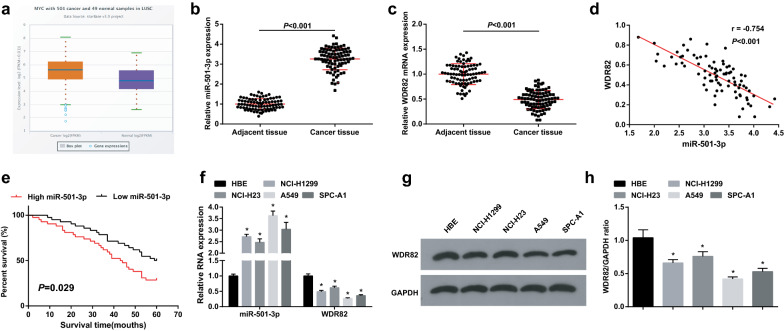
MiR-501-3p is upregulated while WDR82 is downregulated in LC tissues and cells, and high miR-501-3p expression is associated with a poor prognosis. **a** miR-501-3p expression in LC patients detected using Starbase; **b** miR-501-3p expression in LC and adjacent normal tissues detected using RT-qPCR, n = 84; **c** WDR82 expression in LC and adjacent normal tissues detected using RT-qPCR, n = 84; **d** correlation analysis between miR-501-3p and WDR82; **e** the Kaplan–Meier analysis of miR-501-3p in relation to OS rate in LC patients; **f** miR-501-3p and WDR82 expression in LC cell lines and HBE cells detected using RT-qPCR, N = 3; g/h, WDR82 protein expression in LC cell lines and HBE cells detected using Western blot analysis, N = 3; *P < 0.05 vs the HBE cells; the measurement data were expressed as mean ± standard deviation, unpaired t-test was performed for comparisons between two groups, one-way ANOVA was used for comparisons among multiple groups and Tukey’s post hoc test was used for pairwise comparisons after one-way ANOVA

Firstly, miR-501-3p expression in LC patients was analyzed by starbase website, showing an increase in its level (Fig. 1a). miR-501-3p and WDR82 expression in tissues were assessed and we found that LC tissues had higher miR-501-3p expression and lower WDR82 expression versus adjacent normal tissues (Fig. 1b, c), and the Pearson test revealed that there existed a negative relationship between expression of miR-501-3p and WDR82 (Fig. 1d). It was found through the Kaplan–Meier analysis that high miR-501-3p expression was associated with a decreased OS (Fig. 1e). miR-501-3p and WDR82 expression in cells were assessed as well and we found that versus the HBE cells, miR-501-3p was upregulated while WDR82 was downregulated in LC cell lines, especially in A549 and SPC-A1 cells (Fig. 1f–h). Thus, these two cell lines were selected.

### Extraction and identification of M2 exosomes

**Fig. 2:**
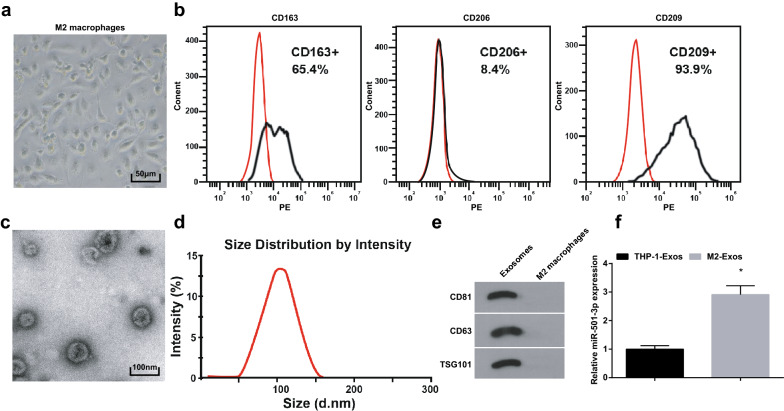
Extraction and identification of M2 exosomes; miR-501-3p expression detection. **a** morphological observation of M2 macrophages; **b** surface marker expression of M2 macrophages detected using flow cytometry; **c** morphological observation of M2 exosomes using a TEM; **d** particle size of exosomes measured by NTA; **e** M2 exosome markers detected using Western blot analysis; **f** miR-501-3p expression in M2 exosomes and THP-1 exosomes was detected using RT-qPCR. *P < 0.05 vs THP-1 exosomes

Treated with LPS and IFN-γ for 24 h, THP-1 cells were stimulated by IL-4 for 72 h and differentiated into M2 macrophages expressing CD206 (M2 macrophage phenotype marker) and not expressing CD163 and CD209. Under a microscope, M2 macrophages were mainly prolate (Fig. 2a, b). It was observed using a TEM that the vesicles separated from M2 macrophages had a bilayer membrane structure, which is a characteristic of exosomes. The shape of exosomes was solid and compact, and they were disc- or cup-shaped (Fig. 2c). The diameter of vesicles was mainly about 100 nm, detected by NTA (Fig. 2d). The results of Western blot analysis showed that there were obvious exosome marker proteins CD68, CD63 and TSG101 in the exosomes, indicating that exosomes were successfully extracted (Fig. 2e). RT-qPCR was utilized to determine miR-501-3p expression in M2 exosomes and THP-1 exosomes and found that it was upregulated in M2 exosomes (Fig. 2f).

### M2 exosomes promote LC cell growth

**Fig. 3:**
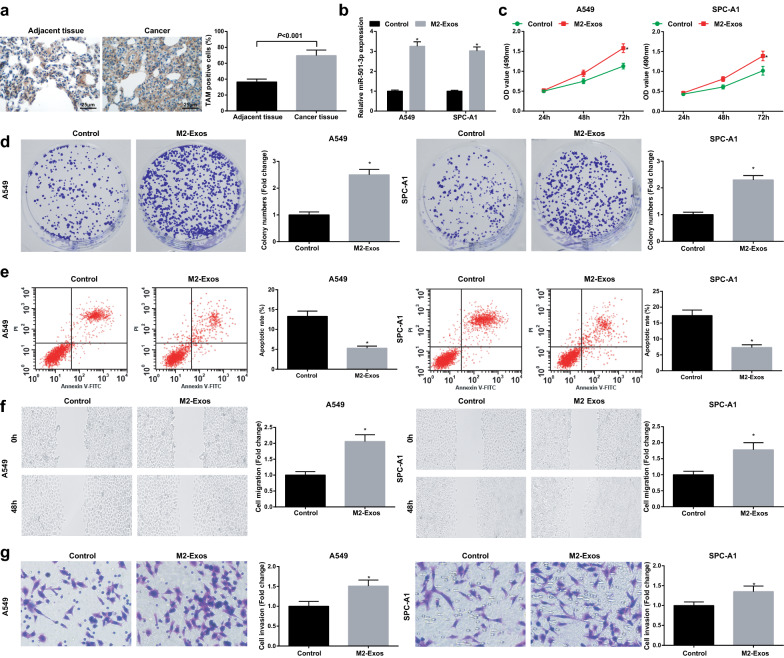
M2 exosomes promote LC cell growth. **a** F4/80 positive expression in LC tissues and adjacent normal tissues was detected using immunohistochemistry; **b** miR-501-3p expression in A549 and SPC-A1 cells detected using RT-qPCR; **c** proliferation of A549 and SPC-A1 cells detected by CCK-8 assay; **d** colony formation ability of A549 and SPC-A1 cells detected by colony formation assay; **e** apoptosis of A549 and SPC-A1 cells detected by flow cytometry; **f** migration ability of A549 and SPC-A1 cells detected by scratch assay; **g** invasion ability of A549 and SPC-A1 cells detected by Transwell assay; N = 3; the measurement data were expressed as mean ± standard deviation and unpaired t-test was performed for comparisons between two groups

M2 exosomes were reported to regulate cancer cell growth by transmitting miRNAs [17, 26]. Immunohistochemistry was performed to test TAM (F4/80 positive) level in LC tissues and adjacent normal tissues. It was suggested TAM level of LC tissues was increased (Fig. 3a). A549 and SPC-A1 cells were treated with M2 exosomes to detect their role in LC cell growth (LC cells without any treatment served as a control). It was discovered through RT-qPCR that the M2 exosomes upregulated miR-501-3p (Fig. 3b). The results of our experiments indicated that M2 exosomes suppressed apoptosis and promoted proliferation, migration and invasion of LC cells (Fig. 3c–g), suggesting the promoting role of M2 exosomes in LC cell growth.

### M2 macrophages-derived exosomal miR-501-3p facilitates LC cell growth

**Fig. 4:**
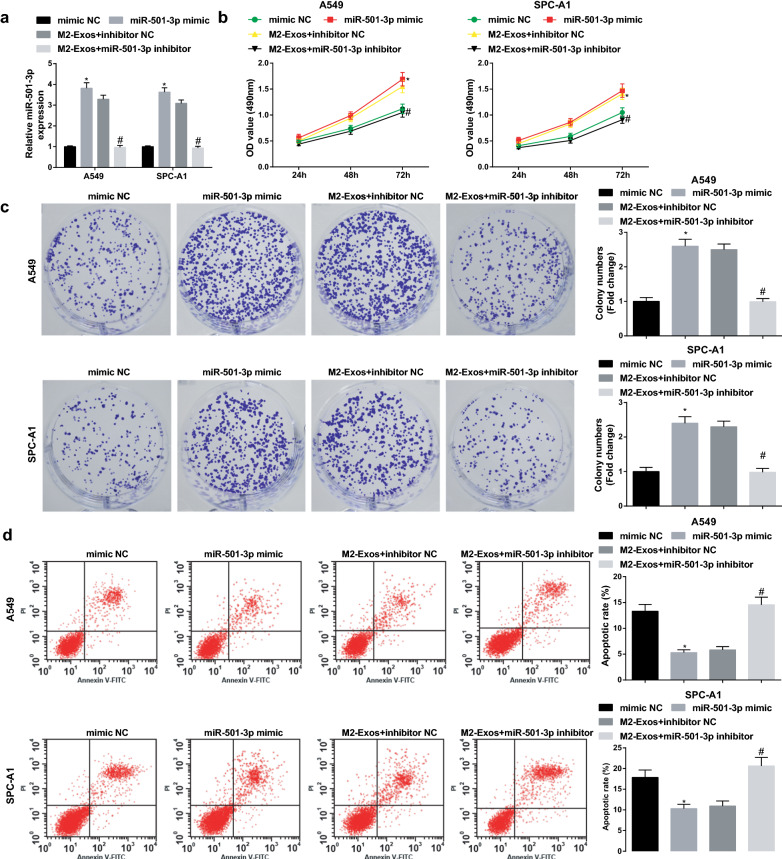
M2 macrophages-derived exosomal miR-501-3p facilitates LC cell proliferation and suppresses apoptosis. **a** miR-501-3p expression in A549 and SPC-A1 cells detected using RT-qPCR; **b** proliferation of A549 and SPC-A1 cells detected by CCK-8 assay; **c** colony formation ability of A549 and SPC-A1 cells detected by colony formation assay; **d** apoptosis of A549 and SPC-A1 cells detected by flow cytometry; N = 3; *P < 0.05 vs the mimic NC group, ^#^*P* < 0.05 vs the M2-Exos + inhibitor NC group; the measurement data were expressed as mean ± standard deviation, one-way ANOVA was used for comparisons among multiple groups and Tukey’s post hoc test was used for pairwise comparisons after one-way ANOVA

**Fig. 5:**
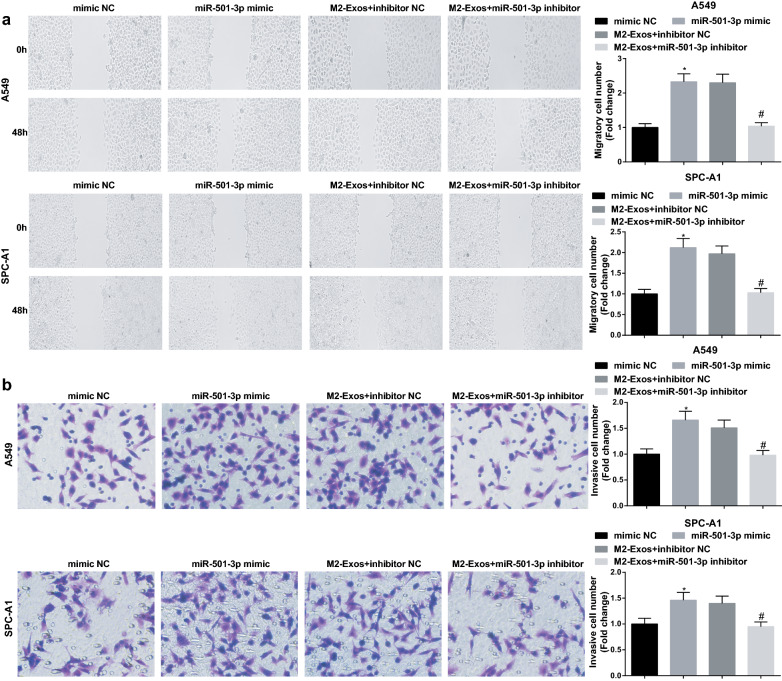
M2 macrophages-derived exosomal miR-501-3p facilitates LC cell invasion and migration. **a** migration ability of A549 and SPC-A1 cells detected by scratch assay; **b** invasion ability of A549 and SPC-A1 cells detected by Transwell assay; N = 3; *P < 0.05 vs the mimic NC group, ^#^*P* < 0.05 vs the M2-Exos + inhibitor NC group; the measurement data were expressed as mean ± standard deviation, one-way ANOVA was used for comparisons among multiple groups and Tukey’s post hoc test was used for pairwise comparisons after one-way ANOVA

The above data implied that M2 exosomes regulated miR-501-3p expression, thus we inferred that miR-501-3p may participate in the biological functions of LC cells. Cells were treated with miR-501-3p mimic or M2 exosomes conveying miR-501-3p inhibitor to figure out its effect on LC cells (Fig. 4a), and the results of our experiments showed that miR-501-3p mimic restricted apoptosis and facilitated proliferation, migration and invasion of A549 and SPC-A1 cells, while these effects could be reversed by M2 exosomes downregulating miR-501-3p (Figs. 4b–d; 5a, b). These results indicated that similar to M2 macrophages, miR-501-3p promoted LC cell growth, whereas this oncogenic impact of miR-501-3p was blocked by M2 macrophages downregulating miR-501-3p.

### MiR-501-3p targets WDR82

**Fig. 6:**
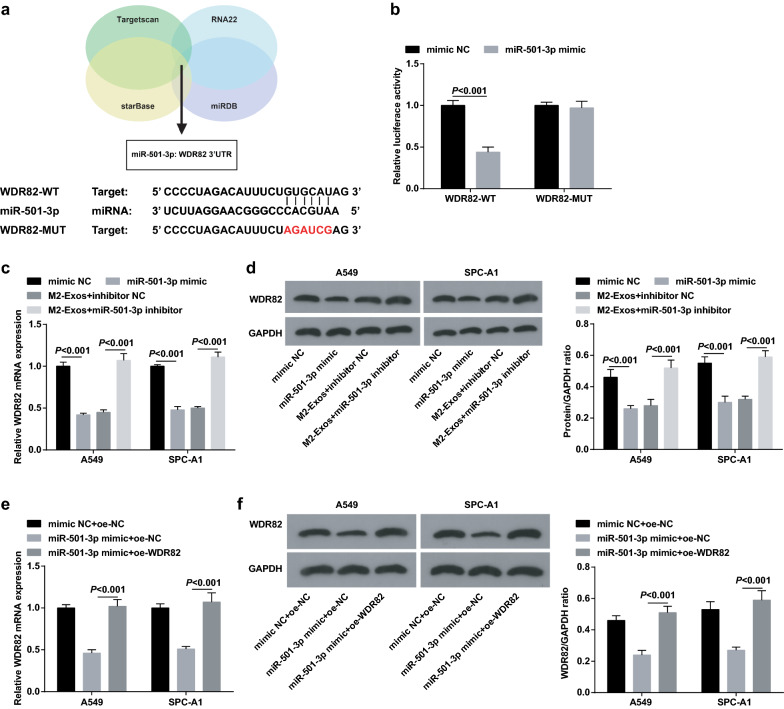
MiR-501-3p targets WDR82. **a** the predicted binding sites of miR-501-3p and WDR82 on Targetscan database; **b** the binding of miR-501-3p to WDR82 was confirmed by dual luciferase reporter gene assay; **c–f** expression of WDR82 in A549 and SPC-A1 cells detected by RT-qPCR and Western blot analysis; N = 3; the measurement data were expressed as mean ± standard deviation, unpaired t-test was performed for comparisons between two groups, one-way ANOVA was used for comparisons among multiple groups and Tukey’s post hoc test was used for pairwise comparisons after one-way ANOVA

It was predicted at Targetscan that there existed binding sites between miR-501-3p and WDR82 (Fig. 6a). It was further confirmed using dual luciferase reporter gene assay that WDR82 was a target gene of miR-501-3p, and the results implied that miR-501-3p mimic lowered the luciferase activity of pGL3-WDR82-WT while had no impact on that of pGL3-WDR82-MUT (Fig. 6b). Results of RT-qPCR and Western blot analysis indicated that miR-501-3p mimic transfection reduced WDR82 expression in LC cells. The same trend was observed in M2 exosomes. In addition, co-culture with M2 exosomes containing miR-501-3p inhibitor, LC cells presented upregulated WDR82. Though transfecting A549 cells and SPC-A1 cells with WDR82 overexpression vector, WDR82 expression was subsequently reduced due to the overexpression of miR-501-3p (Fig. 6c–f).

### MiR-501-3p downregulates WDR82 to accelerate LC cell growth

**Fig. 7:**
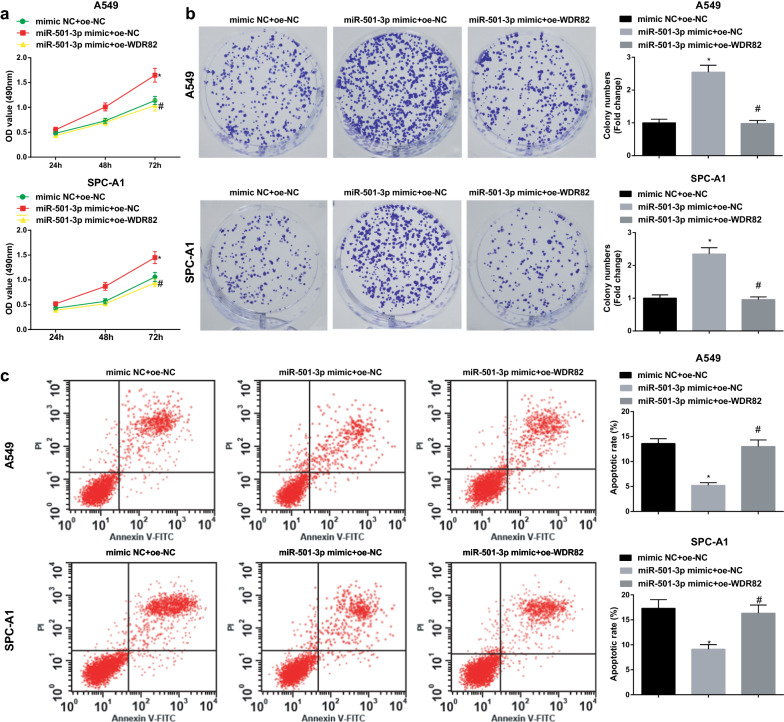
MiR-501-3p downregulates WDR82 to accelerate LC cell proliferation and reduce apoptosis. **a** proliferation of A549 and SPC-A1 cells detected by CCK-8 assay; **b** colony formation ability of A549 and SPC-A1 cells detected by colony formation assay; **c** apoptosis of A549 and SPC-A1 cells detected by flow cytometry; N = 3; *P < 0.05 vs the mimic NC + oe-NC group, ^#^*P* < 0.05 vs the miR-501-3p mimic + oe-NC group; the measurement data were expressed as mean ± standard deviation, one-way ANOVA was used for comparisons among multiple groups and Tukey’s post hoc test was used for pairwise comparisons after one-way ANOVA

**Fig. 8:**
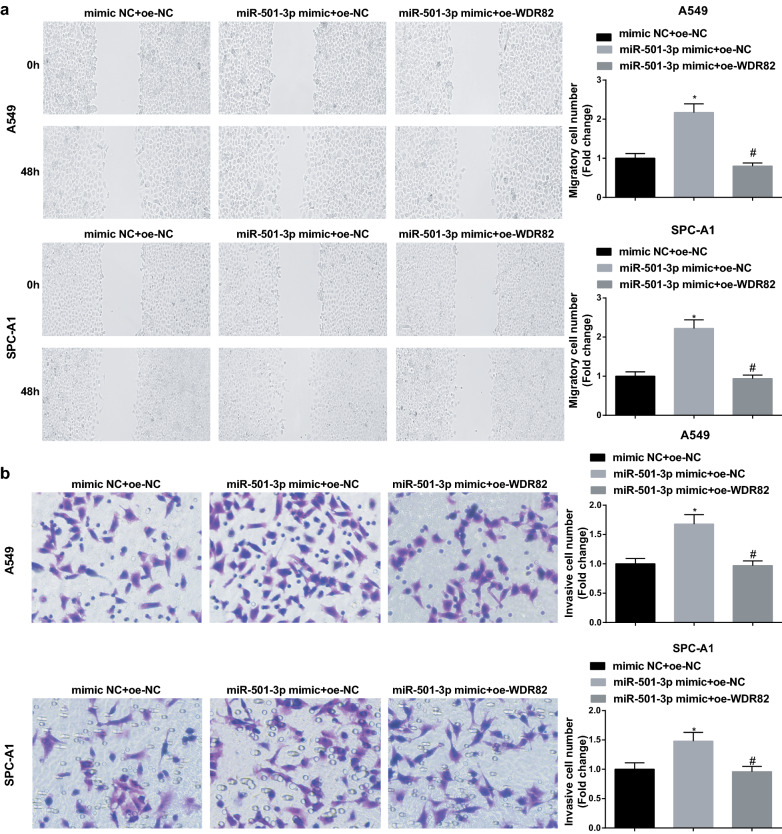
MiR-501-3p downregulates WDR82 to promote LC cell invasion and migration. **a** migration ability of A549 and SPC-A1 cells detected by scratch assay; **b** invasion ability of A549 and SPC-A1 cells detected by Transwell assay; N = 3; *P < 0.05 vs the mimic NC + oe-NC group, ^#^*P* < 0.05 vs the miR-501-3p mimic + oe-NC group; the measurement data were expressed as mean ± standard deviation, one-way ANOVA was used for comparisons among multiple groups and Tukey’s post hoc test was used for pairwise comparisons after one-way ANOVA

To identify whether WDR82 could affect the role of miR-501-3p mimic in LC cells, we conducted rescue assays with miR-501-3p mimic and WDR82 overexpression vector transfection. It was finally found that though miR-501-3p mimic transfection promoted LC cell growth, subsequent transfection of WDR82 overexpression vector would impair the growth of LC cells (Figs. 7a–c; 8a, b).

The above data showed that M2 exosomes transmit miR-501-3p to target WDR82, thus facilitating proliferation, migration and invasion, and restraining apoptosis of LC cells.

## Discussion

LC, one of the main reasons of cancer-related deaths, is still a serious global public health issue to human beings [27]. Exosomes are secreted by various cell types and participate in material transportation and intracellular communication through signaling molecules on cell membrane surface [15]. We aimed to identify the role of M2 macrophages-derived exosomal miR-501-3p during the progression of LC via the regulation of WDR82, and we found that the M2 exosomal miR-501-3p could promote LC cell growth by inhibiting WDR82, thus facilitating LC development.

The lung tumor-exosomes in M2 macrophage polarization has been found to offer a novel therapeutic target for immunotherapy of LC [28]. The LC cells were treated with M2 exosomes to explore the effects of the exosomes on LC cells. The results showed that M2 exosomes promoted malignant behaviors of LC cells. Similarly, Lan et al. have demonstrated that M2 exosomes promote cell migration and invasion in colon cancer [26], and a recent publication has revealed that TAM-derived exosomes facilitate the migration of gastric cancer cells [29]. Moreover, we determined miR-501-3p expression in tissues and cell lines, and it was revealed that miR-501-3p was upregulated in LC tissues and cell lines, respectively versus adjacent normal tissues and human normal bronchial epithelial cells HBE, and M2 exosomes were found to further upregulate miR-501-3p. Consistently, Yin et al. have observed that miR-501-3p is highly expressed in PDAC tissues and TAM-derived exosomes [17], and it has been recently validated that miR-501-5p expression is markedly increased in gastric cancer tissues and cell lines [30]. Chen et al*.* have found that upregulated miR-501-5p is found in patients with lung adenocarcinoma [14]. To further investigate the impact of miR-501-3p on LC progression, the LC cells were transfected with altered miR-501-3p to assess the biological functions of LC cells, and our results showed that the elevation of miR-501-3p decelerated apoptosis and accelerated proliferation, migration and invasion of LC cells. In accordance with this finding, it has been elucidated that miR-501 overexpression promotes cervical cancer cell growth [31], and Yu et al. have verified that the ectopic expression of miR-501 promotes hepatocellular carcinoma cell invasion and epithelial-mesenchymal transition, whereas the low expression leads to the contrary results [32]. In addition, the downregulation of miR-501 has been identified to inhibit malignant episodes of hemangioma cells [33].

Furthermore, miRNAs are known to bind the 3′-untranslated region of mRNA to degrade mRNA and therefore to negatively regulate relevant gene expression [34]. In this present study, we confirmed through bioinformatic prediction and dual luciferase reporter gene assay that there existed a targeting relationship between miR-501-3p and WDR82. Nevertheless, this relationship is still scarcely investigated. The expression of WDR82 was also determined in our study and we found that WDR82 was lowly expressed in LC tissues and cells when compared with adjacent normal tissues and HBE cells. In line with this result, Liu et al. have unraveled that WDR82 expression is obviously decreased in colorectal cancer tissues versus paired noncancerous tissues from patients with colorectal cancer [20]. In view of the abnormal expression of WDR82, we overexpressed it in LC cells to identify its role in LC cell growth, and it was found that the overexpression of WDR82 was able to suppress the malignant episodes of LC cells. However, the anti-tumor effect of WDR82 remains to be further explored due to the limited literature.

## Conclusion

In conclusion, results of our research indicated that M2 exosomal miR-501-3p could promote LC cell growth via targeting WDR82, thus accelerating the progression of LC. This study may provide novel biomarkers for the treatment and diagnosis of LC, while more efforts are still needed.

## Supplementary Information

**Additional file 1: Table S1.** Clinical baseline characteristics of patients.

## Data Availability

Not applicable.

## Abbreviations

miRNAs: MicroRNAs
M2 exosomes: M2 macrophages-derived exosomes
LC: Lung cancer
WDR82: WD repeat domain 82
SCLC: Small cell lung cancer
NSCLC: Non-small cell lung cancer
PDAC: Pancreatic ductal adenocarcinoma
OS: Overall survival
LPS: Lipopolysaccharide
IL-4: Interleukin 4
TEM: Transmission electronic microscope
NTA: Nanoparticle tracking analysis
TSG101: Tumor susceptibility gene 101
NC: Negative control
oe: Overexpressed
CCK-8: Cell counting kit-8
RT-qPCR: Reverse transcription quantitative polymerase chain reaction
