# Supplementary material for: M2 macrophages‐derived exosomal microRNA-501-3p promotes the progression of lung cancer via targeting WD repeat domain 82
Source: Cancer Cell Int. 2021 Feb 5;21:91. doi: 10.1186/s12935-021-01783-5 (PMC7866732; doi:10.1186/s12935-021-01783-5)
Supplement: Supplementary file 1 — Additional file 1: Table S1. Clinical baseline characteristics of patients. [file 12935_2021_1783_MOESM1_ESM.docx]

**Additional file 1: Table S1** Clinical baseline characteristics of patients

| Clinicopathological characteristics | Cases |
| --- | --- |
| Gender |  |
| Male | 57 |
| Female | 27 |
| Age |  |
| ＞ 50 | 35 |
| ≤ 50 | 49 |
| TNM stage |  |
| Ⅰ ＋ Ⅱ | 52 |
| Ⅲ ＋ Ⅳ | 32 |
| Pathological type |  |
| Squamous cell carcinoma | 24 |
| Adenocarcinoma | 11 |
| Small cell lung cancer | 28 |
| Non-small lung cell cancer | 21 |
